# Salinity Adaptation and the Contribution of Parental Environmental Effects in *Medicago truncatula*

**DOI:** 10.1371/journal.pone.0150350

**Published:** 2016-03-04

**Authors:** Ken S. Moriuchi, Maren L. Friesen, Matilde A. Cordeiro, Mounawer Badri, Wendy T. Vu, Bradley J. Main, Mohamed Elarbi Aouani, Sergey V. Nuzhdin, Sharon Y. Strauss, Eric J. B. von Wettberg

**Affiliations:** 1 Department of Plant Pathology, University of California Davis, Davis, California, United States of America; 2 Molecular and Computational Biology, University of Southern California, Los Angeles, California, United States of America; 3 Department of Plant Biology, Michigan State University, Lansing, Michigan 48824, United States of America; 4 Plant Cell Biotechnology, Instituto de Tecnologia Química e Biológica, Universidade Nova de Lisboa, Oeiras, Portugal; 5 Centre of Biotechnology of Borj Cedria, B.P. 901, Hammam-Lif, Tunisia; 6 Department of Evolution and Ecology and Center for Population Biology, University of California Davis, Davis, California, United States of America; 7 Department of Biological Sciences and International Center for Tropical Botany, Florida International University, Miami, Florida, United States of America; 8 Kushlan Institute for Tropical Science, Fairchild Tropical Botanic Garden, Coral Gables, Florida, United States of America; Institute for Sustainable Plant Protection, C.N.R., ITALY

## Abstract

High soil salinity negatively influences plant growth and yield. Some taxa have evolved mechanisms for avoiding or tolerating elevated soil salinity, which can be modulated by the environment experienced by parents or offspring. We tested the contribution of the parental and offspring environments on salinity adaptation and their potential underlying mechanisms. In a two-generation greenhouse experiment, we factorially manipulated salinity concentrations for genotypes of *Medicago truncatula* that were originally collected from natural populations that differed in soil salinity. To compare population level adaptation to soil salinity and to test the potential mechanisms involved we measured two aspects of plant performance, reproduction and vegetative biomass, and phenological and physiological traits associated with salinity avoidance and tolerance. Saline-origin populations had greater biomass and reproduction under saline conditions than non-saline populations, consistent with local adaptation to saline soils. Additionally, parental environmental exposure to salt increased this difference in performance. In terms of environmental effects on mechanisms of salinity adaptation, parental exposure to salt spurred phenological differences that facilitated salt avoidance, while offspring exposure to salt resulted in traits associated with greater salt tolerance. Non-saline origin populations expressed traits associated with greater growth in the absence of salt while, for saline adapted populations, the ability to maintain greater performance in saline environments was also associated with lower growth potential in the absence of salt. Plastic responses induced by parental and offspring environments in phenology, leaf traits, and gas exchange contribute to salinity adaptation in *M*. *truncatula*. The ability of plants to tolerate environmental stress, such as high soil salinity, is likely modulated by a combination of parental effects and within-generation phenotypic plasticity, which are likely to vary in populations from contrasting environments.

## Introduction

Plants experience a range of environmental stresses from different sources (e.g., drought, heavy metals, low-nutrient soils) and of varying magnitudes [1–2]. The ability of some plants to maintain vegetative growth and reproduction despite environmental stress suggests that adaptations can evolve within populations. These responses can be genetically fixed within a population resulting in local adaptation. Additional adaptive responses can be triggered by environmental conditions experienced during the parental (i.e., parental environmental effects) and offspring’s (i.e., phenotypic plasticity) generations [3–10].

In terrestrial plants, salt stress occurs over an estimated 6% of all lands and approximately 20% of irrigated agricultural lands [11]. Such levels are predicted to increase due to both natural processes (e.g., proximity of saline groundwater, sea-level rise, leaching from rocks, dispersal of oceanic salts via wind and precipitation) and agricultural practices (e.g., irrigation, removal of deep rooted native vegetation; see references in [11]). Soil salinity is likely to vary spatially and temporally [12] and its distribution pattern will influence adaptive responses of plant populations [13–15]. Consequently, when soil salinity levels differ between habitats, each population may evolve different strategies. A long history of studies demonstrates plant local adaptation to a range of environmental stressors (see references in [16–17]), though the mechanisms through which adaptation is achieved vary depending on the spatial and temporal heterogeneity of the stress. For example, if stressors vary on a smaller spatial scale relative to the range of offspring dispersal, genotypes that have the ability to adjust their phenotypes to match their environment may be favored, i.e., plasticity may evolve in these populations [13–15]. Alternatively, when the stressors vary on a larger scale than the range of offspring dispersal, favored alleles may become fixed within populations resulting in population specific adaptations to their home environment [14].

Empirical studies testing adaptive hypotheses typically use seeds from parental plants grown in a single environment to control for parental environmental effects (e.g., following advice such as that by [18]), potentially masking important mechanisms of adaptive response. Parental environmental effects, which include maternal and paternal environmental effects, are a form of transgenerational plasticity where the environment in which seeds develop influences the offspring’s phenotype [8–9, 19–20]. Parental environmental effects were once considered a source of noise in empirical studies (e.g., [19]). However recent studies show that the impact of parental environmental experiences on offspring phenotypes varies among genotypes [20–23] and that these differences can be favored by natural selection [5, 22–25]. Thus rather than ‘controlling’ parental environment, studies should incorporate these effects into empirical tests of adaptation.

Parental environmental effects have been shown to contribute to salinity adaptation. For example, *Iris hexagona* seeds developed in saline parental conditions germinated earlier when exposed to salt [26], and *Sorghum bicolor* offspring from salt-treated parents excluded salt upon exposure [27]. Salinity tolerance of *Arabidopsis thaliana* increased with the number of generations grown in saline soils [22]. These findings highlight the importance of parental environmental effects on salinity adaptation but, with the exception of a few studies like Suter and Widmer [22], reports on whether the effects of parental salinity persist beyond the offspring’s seedling stage through to offspring performance are rare.

Salinity stress occurs through osmotic stress and ion toxicity, and mechanisms of salinity adaptation fall along a gradient from avoidance to tolerance [11]. Avoidance mechanisms prevent exposure, while tolerance mechanisms allow individuals to maintain growth in the presence of high soil salinity levels; and both may contribute to salinity adaptation. These complementary mechanisms for coping with abiotic stress are similar to the dual strategies of induced and constitutive defenses to plant herbivory [28]. In general, avoidance mechanisms may include seed dormancy or faster plant growth, with both germination and flowering being critical life stages [11, 29–32]. Germination is the one of the earliest stages an offspring can experience environmental stress and seeds can either geminate or remain dormant until conditions become more favorable (e.g., [33–34]). Age at flowering is an important transition stage of the life cycle, and multiple studies on adaptation to stress have demonstrated that selection favors earlier flowering in stressful environments [30, 32, 35], including soil salinity (e.g., [23]). Recent field and greenhouse studies of *Medicago truncatula* demonstrated selection favors earlier flowering in saline environments [36].

Salinity and drought tolerance mechanisms are similar [11, 37] and can involve leaf, stem, and root traits. Salinity stress typically results in plants developing small leaf lamina area and decreased stomatal opening [37]. Salinity tolerance mechanisms can include sequestering of Na+ ions in vacuoles, increasing leaf tissue water content, producing osmolytes, increasing water use efficiency [11, 37], and raising chlorophyll concentrations in the mesophyll [38–39]. Nevertheless, these physiological responses can result in costs to carbon acquisition, resulting in decreased growth rates in the absence of salt [40]. In non-saline environments, we expect traits that confer greater plant growth to be favored, such as larger leaf lamina area, greater carbon acquisition rates, and greater leaf production [41]. Thus, different phenological, morphological, and physiological trait values may have evolved in populations occurring in saline and non-saline environments.

In this study, we quantify the influence of soil salinity on offspring whose parents were grown under experimentally controlled saline and non-saline conditions on the annual *M*. *truncatula* var. *truncatula* Gaertn (Fabaceae) from four natural populations; two from saline environments and two from non-saline environments. Thus data gathered from this experiment are from plants that have undergone two generations of salinity treatments (i.e., 0 mM NaCl or 100 mM NaCl treatments during the parental and offspring generation). We focus on population responses to soil salinity including the rarely tested role of parental environmental effects of soil salinity on plant performance. Populations of each soil origin are expected to be adapted to their native soil salinity level (i.e., saline or non-saline, [36, 42]). Generally, this species is predominantly found in non-saline areas (unpubl. data of the authors); molecular markers also show extensive gene flow between saline and non-saline areas [36, 42]. Despite extensive gene flow, there is evidence for adaptation to salinity in these natural populations [36]. We hypothesize that spatial heterogeneity in abiotic stress within seed dispersal distances should favor plasticity as a mode of adaptation [13–15]. We specifically test the genetic and environmentally basis of two mechanisms of salinity adaptation: salinity avoidance via adjustments in phenology (i.e., germination and flowering time) and salinity tolerance via leaf traits associated with plant growth (i.e., leaf area, leaf numbers, leaf water content, and carbon acquisition rates).

## Materials and Methods

*Medicago truncatula* is an annual plant with high selfing rates [43] commonly found throughout the Mediterranean region [44]. It is an emerging model organism with a completed and well-annotated genome sequence, extensive mutant collections, and growing numbers of wild collected ecotypes [45–46]. We used a genotype collection originating from two populations occurring on coastal saline soils (TN1 and TN8) and two populations occurring on non-saline soils (TN7 and TN9) in Northern Tunisia [47]. Soil salinity is approximately eight-times greater at the two saline populations (6.5 g/L) than at the two non-saline populations (0.8 g/L; [47]). Throughout the text, we categorize each population’s home soil salinity level as either saline or non-saline. In saline environments, winter rains decrease soil salinity levels; when rains cease the salt concentrations increase coinciding with flowering of natural populations [36]. Both saline populations occur within a km of potentially suitable non-saline habitats, while both non-saline populations are at least 25 km from the nearest saline habitat (the authors, unpublished data).

The original collection consisted of 15 genotypes for each population [47] from which we randomly selected 10 genotypes from all populations except for TN7 that was represented by nine genotypes (S1 Table). An additional advantage of this collection is that all genotypes were propagated for three generations via single seed descent in 8L pots under greenhouse non-saline conditions at the Centre for Biotechnology, Borj Cedria, Tunisia prior to this study.

### Salinity experiment parental and offspring generation

During the parental generation, seeds of the 39 genotypes were scarified to induce germination and were individually planted into 656 mL pots containing a 2:1 mixture of sterilized horticultural sand:soil mix at an UC Davis greenhouse in May 2009. Two weeks after germination, salinity treatments (i.e., parental environments) were imposed by treating plants bi-weekly with a Fahräeus nutrient solution modified to contain either 0 mM NaCl or 100 mM NaCl (following [48]). The salt concentration used was similar to the salinity observed in the field to cause salt stress but not extreme mortality in *M*. *truncatula* [48]. Pods were collected as they matured until the end of the experiment. Seeds used for the second generation were from pods collected during the peak seed maturation period and a subset of seeds was weighed for each genotype before the second-generation experiment (i.e., offspring environments).

For the offspring generation, 40 seeds for each genotype and parental environment combination were scarified to induce germination and individually planted into 164 mL pots filled with 2:1 sand:soil mix during January 2010. Each genotype had 10 replicates for each parental and offspring environment combination with the exception of the TN9.20 genotype, which had at least five replicates. Pots were fully randomized across racks and placed in an open field next to the UC Davis’ greenhouses to allow for natural variation in temperature and precipitation from January 1st to August 1st 2010. Once seeds were sown, salinity treatments were applied bi-weekly by adding 100 mL of Fahräeus solution mixed with 0 mM or 100 mM NaCl (i.e., offspring environment) to each pot. A seed was considered germinated when cotyledons were visible. The number of days since the seed was sown was recorded. Non-germinated seeds were checked for viability and replaced with seedlings that were germinated in Petri dishes under non-saline conditions; replacement occurred within the first seven days of the experiment to give a total of 1552 pots.

Traits related to mechanisms of salinity avoidance (phenological traits: number of days to germination and flowering) and tolerance (traits associated with plant growth: number of leaves, leaf area (cm^2^), leaf water content (mg/mg), and instantaneous carbon acquisition rate per leaf area (μmoles CO_2_/cm^2^) were measured. After three months, at the start of flowering, three random plants were harvested per parental and offspring environment combination for each genotype for a total of 457 plants. Leaf area was measured for the three most recent fully developed leaves to the nearest 0.1 cm^2^. Plants were dried to a constant weight, reweighed, and total vegetative dry biomass (g) was recorded.

Instantaneous carbon acquisition rates (μmoles CO_2_/cm^2^) were measured on four genotypes per saline origin population and three genotypes per non-saline origin population (S1 Table). For each genotype, three plants were randomly chosen per parental and offspring environment combination for a total of 166 plants using the LiCor 6400 portable photosynthesis system (Li-Cor, Inc., Lincoln, NE) at constant CO_2_ concentration (380 ppm), leaf temperature (26.0°C), relative humidity (38%), saturating photon flux density (1000 μmol quanta/m^2^s), flow rate (500 μmol/s), and leaf-to-air vapor pressure difference (1.0 kPa). Carbon acquisition rates were measured on the most recent fully developed leaf and are reported on a per leaf area basis. Measurements were made between 10am and 4pm during full natural daylight over a three-day period.

The remaining plants were surveyed twice a week to record their age of first flowering and collect matured pods. Mature pods were collected for two months after the initial plant harvest, at which point plants appeared pot bound and the experiment was terminated. Reproduction was quantified as the number of mature pods collected per plant.

### Data analysis

All analyses were performed in SAS v 9.3 [49] and only offspring generation data (S1 File) were included in the analyses (See [36] for parental generation results). Mixed-model ANOVAs using TYPE III sum of squares (PROC MIXED) were used to test whether populations are adapted to their home soil salinity levels and the contribution of parental environment on populations response to salinity. Tests of salinity adaptation were performed using two measures of performance (i.e., dry vegetative biomass, reproduction). For each mixed-model ANOVA, soil origin, population nested within soil origin, parental and offspring environment, and all interactions with these factors were treated as fixed effects. Genotype nested within soil origin and population was treated as a random effect. Significance of the random effect (i.e., genotype and population) was calculated by quantifying the difference in -2 log likelihood scores for the full model and the model excluding the random effects term using a chi-square table with 1 degree of freedom [50]. To meet model assumptions of normality and homoscedasticity, dry vegetative biomass was natural logarithm transformed and number of pods was square root transformed.

A significant origin by offspring environment interaction would indicate that populations originating from each soil salinity level are adapted to their home soil level when each soil origin has greatest performance in its native salinity level. A significant three-way interaction among origin, parental and offspring environment would indicate that parental environmental effects influences population’s salinity adaptation. In order to directly test differences between levels in each class variable in the mixed-model ANOVA, least square means comparisons (LSMEANS) were calculated and reported throughout the results. For ease of interpretation, back transformed means and standard errors are reported in text.

The same mixed-model ANOVAs were used to quantify the effects of soil origin, population, parental environment, and offspring environment on traits associated with salinity avoidance and tolerance. We considered two phenological traits associated with salinity avoidance: germination, flowering; and four morphological and physiological traits associated with salinity tolerance: carbon acquisition rate, leaf number, leaf area, leaf water content. Data were natural logarithm or square root transformed when needed to meet model assumptions of normality and homoscedasticity. Analysis on carbon acquisition rate included the covariate of day of measurement because it had a large effect on measurements.

## Results

**Fig 1:**
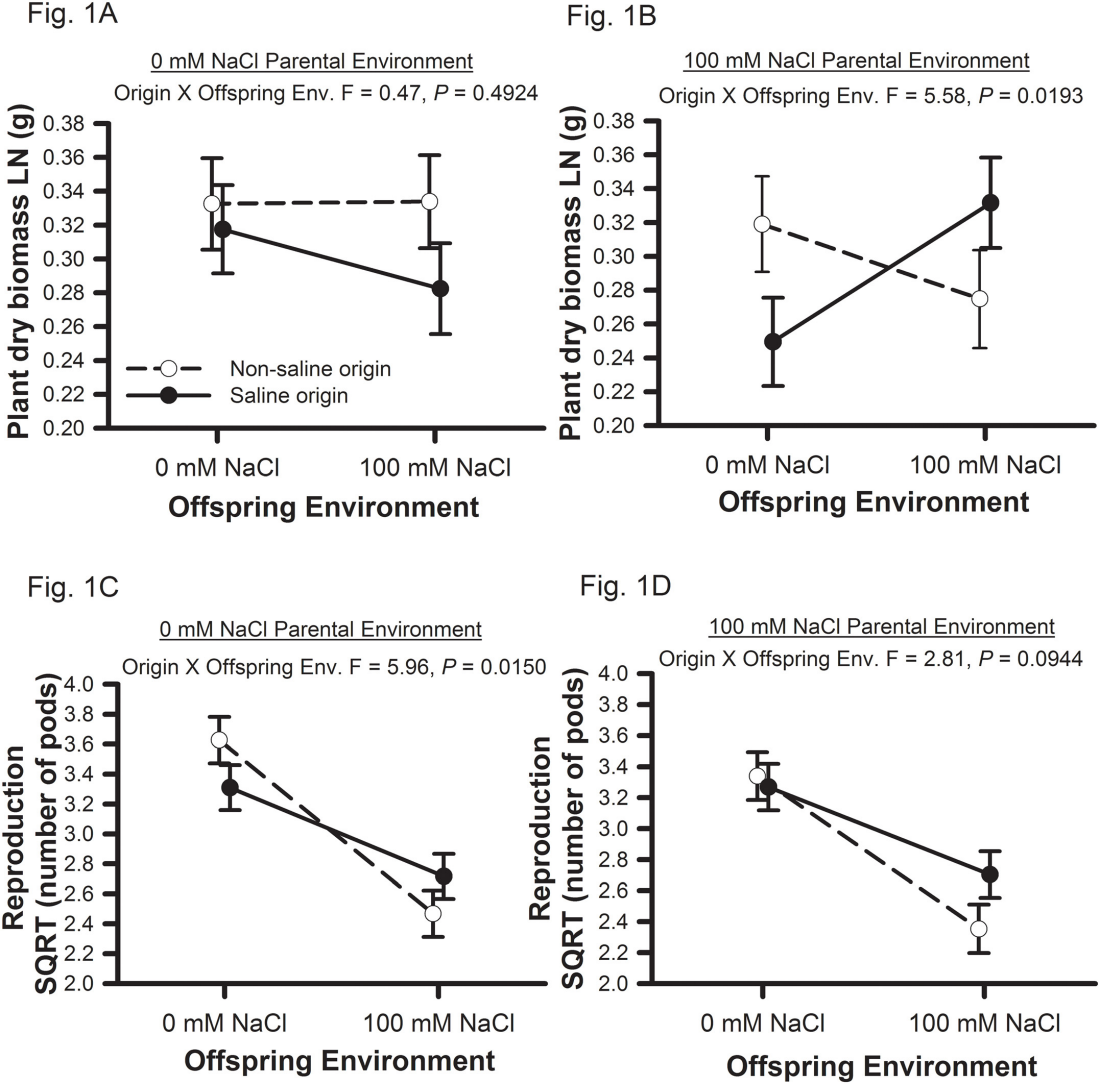
Origin of population performance responses to parental and offspring soil salinity treatments. Origin of population means and one standard error for total dry vegetative biomass (A, B) and reproduction (C, D). Figs A and C show results when parental plants were grown in 0mM NaCl, and Figs B and D show results when parental plants were grown in 100mM NaCl. Non-saline origins are shown with open circles and saline origins are shown with closed circles. ANOVA statistics for origin by offspring environment calculated for each parental environment are shown.

**Fig 2:**
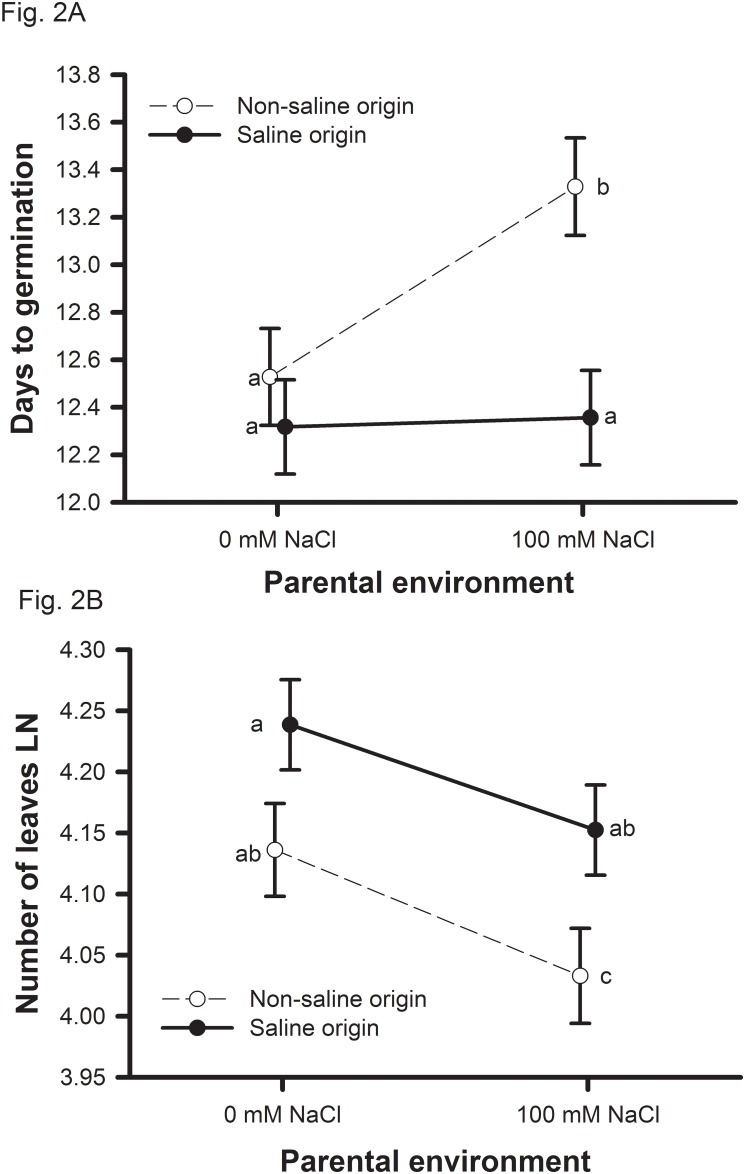
Origin of population parental environmental effects on offspring traits. Origin of population means and one standard error by population for significant effects found for (A) days to germination, (B) number of leaves. Non-saline origins are shown with open circles and saline origins are shown with closed circles. Symbols with different letters indicate which means are significantly different at *P* < 0.05.

**Fig 3:**
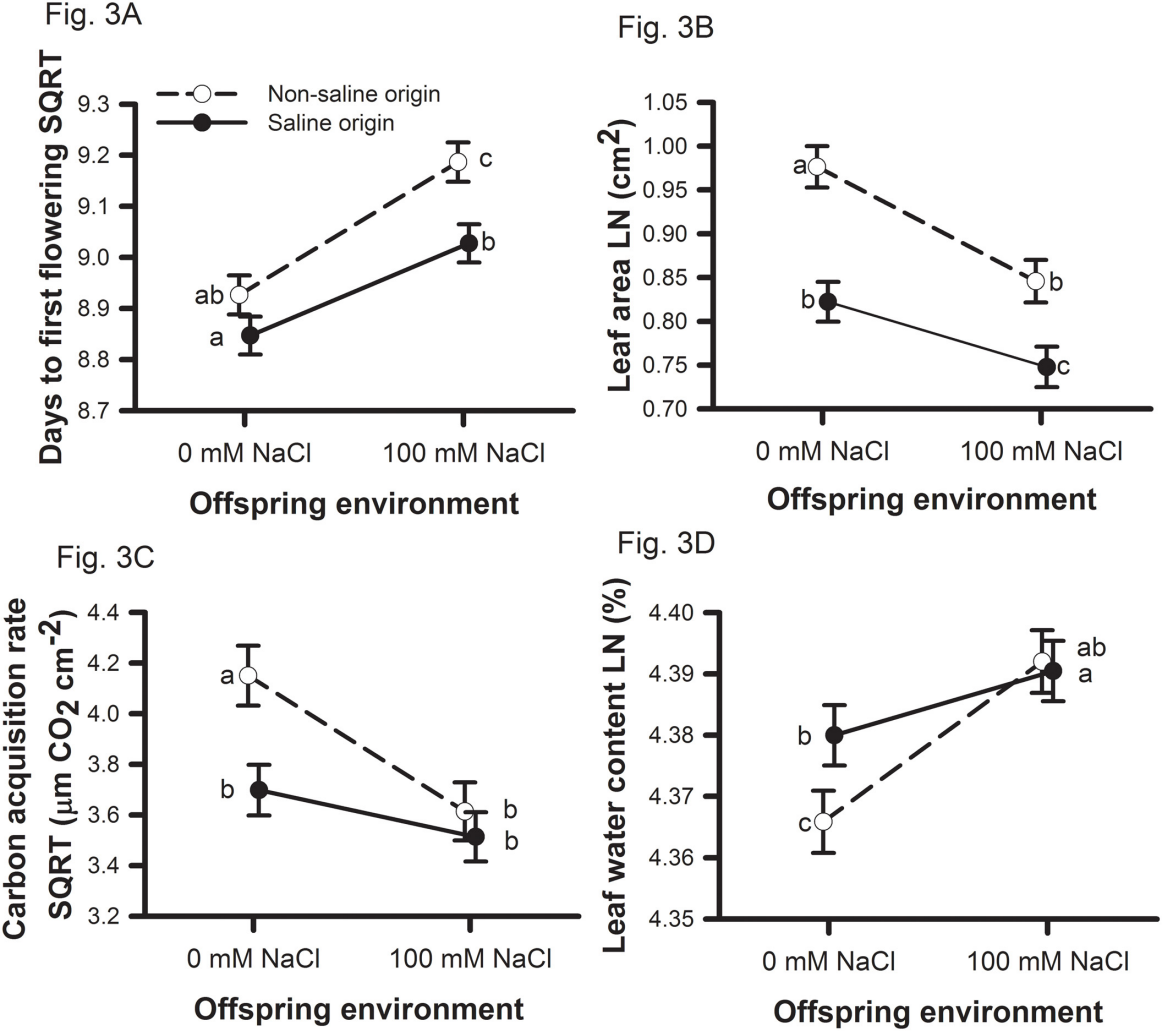
Origin of population offspring environmental effects on offspring traits. Origin of population means and one standard error for significant effects found for (A) days to first flowering, (B) leaf area, (C) carbon acquisition rate, and (D) leaf water content. Non-saline origins are shown with open circles and saline origins are shown with closed circles. Symbols with different letters indicate which means are significantly different at *P* < 0.05.

Soil salinity concentrations (0 or 100 mM NaCl) were manipulated during the parental and offspring generations for 39 genotypes of *Medicago truncatula* originally collected from two saline and two non-saline environments in Tunisia. Two measures of plant performance (i.e., biomass, reproduction) support the hypothesis that populations are adapted to their home soil salinity levels (Table 1; Figs 1 and 2). Salinity adaptation was influenced by parental environment with saline origin plants expressing significantly greater vegetative biomass under salt only when the parent generation also experienced saline conditions (Fig 1A and 1B). Salinity adaptation was likely conferred by both avoidance and tolerance mechanisms (Table 2, Figs 2 and 3). Consistent with expectations that earlier completion of the life cycle is favorable in saline environments, saline origin plants germinated (Fig 2A) and reproduced (Fig 3A) earlier than non-saline origin plants when growing in saline environments. We also observed that in the presence of salt, saline origin plants have traits associated with greater salinity tolerance [i.e., maintain leaf number (Fig 2B), leaf water content (Fig 3D), and carbon acquisition rates (Fig 2C)] relative to non-saline origin plants. Non-saline origin plants have traits associated with greater growth potential in the absence of salt [i.e., larger leaf lamina (Fig 3B) and greater carbon acquisition rates (Fig 2C)] relative to saline origin plants.

**Table 1:** Mixed-model analysis of variance results on the performance traits.

Source	df	Total dry biomass LN (g)	Pod production SQRT (counts)
Sample size		443	1095
Origin	1	0.90	0.11
Population(Origin)	2	0.73	14.94****
Parental Environment	1	1.49	1.85
Offspring Environment	1	0.00	97.33****
Origin × PE	1	0.52	1.08
Origin × OE	1	1.45	8.69**
Population(Origin) × PE	2	0.74	0.38
Population(Origin) × OE	2	2.80†	2.05
PE × OE	1	0.92	0.37
Origin × PE × OE	1	4.75*	0.19
Population(Origin) × PE × OE	2	2.18	0.48
Genotype(Origin Population)	1	0.80	43.60****

Origin of population, population nested within origin of population, parental environment (PE) and offspring environment (OE) were treated as fixed effects and F-values are reported. Genotype was treated as a random effect and Chi-square values are reported.

^†^ 0.10>*P*>0.05,

* *P* < 0.05,

**P < 0.01,

***P < 0.001,

****P < 0.0001.

**Table 2:** Results of mixed-model analysis of variance results on traits associated with mechanisms of salinity avoidance and tolerance.

Source	Days to germination RAW	Days to flowering SQRT	Leaf number LN	Leaf area LN	Leaf water content LN	Carbon acquisition rate SQRT
Sample size	1401	1408	457	451	453	166
Origin	4.65*	5.45*	6.14*	20.70****	0.97	4.55*
Population(Origin)	0.63	1.28	0.29	0.23	0.98	0.81
Parental Environment	24.30****	7.60**	10.67**	0.39	0.08	0.87
Offspring Environment	3.10†	174.50****	1.26	31.32****	35.88****	7.70**
Origin × PE	19.97****	3.22†	0.08	0.42	0.00	0.20
Origin × OE	0.65	5.77*	0.85	2.34	6.56*	1.79
Pop(Origin) × PE	2.60†	0.57	0.33	0.34	0.95	0.71
Pop(Origin) × OE	0.24	3.27*	1.55	0.09	0.03	1.28
PE × OE	1.63	0.51	0.24	2.29	0.09	1.47
Origin × PE × OE	0.00	1.93	0.00	0.04	0.45	1.76
Pop(Origin) × PE × OE	1.52	1.78	1.16	0.36	0.04	0.56
Genotype(Origin Pop)	208.8****	186.9****	15.8****	13.9****	52.9****	3.1†

Origin of population, population, parental environment (PE) and offspring environment (OE) were treated as fixed effects and F-values are reported. Genotype was treated as a random effect and Chi-square values are reported.

^†^ 0.10>*P*>0.05,

* *P* < 0.05,

**P < 0.01,

***P < 0.001,

****P < 0.0001.

### Plant performance influenced by origin of population, parental and offspring environment

Plants from saline and non-saline origins displayed adaptation to their home soil salinity levels as indicated by significant origin by offspring environment interactions for both measures of performance (Table 1; Fig 1). This interaction was dependent upon parental environment for vegetative biomass, but not for reproduction (Table 1; Fig 1). When parental plants of both origins were grown in 0mM NaCl, the origin by offspring environment did not approach significance (Fig 1A), but when parental plants were grown in 100mM NaCl, the origin by offspring environment interaction was significant (Fig 1B). When parental plants were raised in 100mM NaCl, non-saline origin plants had greater biomass relative to saline origin plants when grown in a 0mM NaCl offspring environment. Furthermore, plants from saline origin populations had greater vegetative biomass relative to plants from non-saline origin populations only when both parental and offspring generations were grown under saline conditions (Fig 1A and 1B). The changes in vegetative biomass observed owing to parental environments were not due to maternal provisioning of offspring, as salinity treatments did not influence seed mass in either generation (S2 Table).

In contrast to vegetative biomass, reproduction was not affected by parental environment, but was strongly decreased by saline offspring environment in both population origins (Table 1; Fig 1C and 1D). Plants produced 42.8% fewer pods when growing in 100 mM NaCl [6.5 Pods, SE (-0.4, 0.5)] versus 0 mM NaCl [11.5 Pods, SE (-0.6, 0.6)] offspring environment. The significant origin by offspring environment effect on reproduction, along with the saline and non-saline origin reaction norms crossing (Fig 1C and 1D) indicate that populations are adapted to their home soil salinity level. However, when we tested for the origin by offspring environment interaction within each parental environment, the interaction was significant when parents were grown in 0mM NaCl (Fig 1C), and marginally significant when parents were grown in 100mM NaCl (Fig 1D). Furthermore, we found marginal support that saline and non-saline origins significantly differed in reproduction within either offspring environment. Although plants from both saline and non-saline populations were negatively affected by salt, all tended to perform better in their home soil salinity level (not with statistical significance; Fig 1C and 1D). Within the 0mM NaCl offspring environment, plants from non-saline populations produced 12.1% more pods compared to plants from saline populations (t = 1.07, *P* = 0.2830; Fig 1C and 1D). Within the 100mM NaCl offspring environment, plants from saline populations produced 26.7% more pods compared to non-saline genotypes (t = 1.66, *P* = 0.0978; Fig 1C and 1D).

### Saline origin genotypes germinate and flower earlier than non-saline origin genotypes, mediated by parental environment

Earlier germination and flowering in saline environments is consistent with the evolution of salinity avoidance mechanism. Germination timing was influenced by parental environment, while flowering time was influenced by both parental and offspring environments (Table 2).

The patterns of germination timing for each origin of population were consistent with salinity avoidance, i.e., saline origin plants germinate earlier than non-saline origin plants. Seeds from saline origin populations maintain early germination when seeds were matured on plants from either 0 or 100mM NaCl (Fig 2A; t = 0.33, *P* = 0.7388). Seeds from non-saline origin populations germinated 6.0% later when seeds were matured on plants grown in 100mM NaCl relative to plants grown in 0mM NaCl (Fig 2A; t = 6.51, *P* < 0.0001).

Early flowering is a potential mechanism for accelerating reproduction relative to the increasing salinity levels when winter rains cease. In general, flowering time was delayed when either parents or offspring where raised in 100mM NaCl versus 0mM NaCl (Table 2). However, the delay in flowering when exposed to saline conditions was greater when salinity was experienced during the offspring generation (4.8% delay) versus the parental generation (1% delay). Consistent with the expectation of evolved salinity avoidance in saline origin populations, plants from saline origin populations flowered earlier when growing in 100mM NaCl relative to plants from non-saline origin populations (Fig 3A). The two origin of populations did not differ in flowering time when plants were growing in 0mM NaCl (Fig 3A). The delay in flowering time of non-saline origin populations when growing in 100mM NaCl was strongly influenced by one non-saline population (TN9), which had a greater delay in flowering when growing in 100mM NaCl relative to the other non-saline population (TN7; S1 Fig).

### Saline origin genotypes display traits associated with greater salinity tolerance than non-saline origin genotypes

In addition to traits associated with mechanisms of salinity avoidance, saline populations expressed traits associated with mechanisms of salinity tolerance compared to non-saline populations. Plants from saline origin populations produced 10.6% more leaves (Fig 2B) and displayed traits associated with greater water use efficiency [15.1% smaller leaf area (Fig 3B); 13.9% lower carbon acquisition rate (Fig 3C)] relative to plants from non-saline origin populations (Table 2).

In general, saline environments resulted in negative effects on traits associated with plant growth potential. For example, plants from both origins whose parents were grown in 100mM NaCl resulted in offspring having 10.2% fewer leaves then when parents were grown in 0mM NaCl (Table 2; Fig 2B). Soil salinity experienced during the offspring generation did not influence leaf number but resulted in plants with lower growth potential (Table 2). Plants from both origins that were grown in the 100mM NaCl offspring environments produced 10.8% smaller leaves (Fig 3B) that had 21.3% lower carbon acquisition rates per leaf area (Fig 3C) relative to plants in the 0mM NaCl offspring environments. Plants from non-saline origins had a greater increase in leaf water content in response to the 100mM NaCl offspring environment relative to plants from saline origins (Fig 3D). This was due to saline origin plants having greater leaf water content versus non-saline origin plants when growing in the 0mM NaCl offspring environment (Fig 3D).

All leaf traits displayed significant genetic variation within populations, except for carbon acquisition rate (within population *P* = 0.0782) that displayed genetic variation between origins of populations (Table 2).

## Discussion

Soil salinity had a strong negative influence on performance of *Medicago truncatula* indicating that plants from these populations are not able to completely buffer against salinity stress. However, based on both vegetative biomass and reproduction, populations originating from saline and non-saline environments in northern Tunisia tended to do better in their home soil salinity levels (Fig 1). While the origin of populations does not meet the strict requirements for local adaptation to soil salinity (51), the patterns of performance indicate that saline and non-saline populations harbor alleles favorable in their home environment and that the expression of such alleles may be dependent upon parental environments (Fig 1). The contribution of parental environment to salinity adaptation differed between vegetative biomass and reproduction (Table 1). The signal of adaptation to native salinity levels for vegetative biomass was only expressed when parent plants were raised in saline soils (Fig 1B), while adaptation was not expressed when parent plants were raised in non-saline soils (Fig 1A). In contrast, for reproduction, population adaptation to their native salinity levels was expressed regardless of the salinity of the parental environment (Fig 1C and 1D). We found evidence that both avoidance (Figs 2A and 3A) and tolerance (Figs 2B, 3B and 3C) mechanisms have evolved in saline origin populations. Furthermore, non-saline origin populations have evolved traits associated with greater growth potential (Fig 3B and 3C). Phenological traits were largely influenced by parental environment, while leaf traits were largely influenced by offspring environment (Table 2). Below we focus the discussion on the role of parental and offspring environments on salinity adaptation and mechanisms of salinity adaptation.

### Parental and offspring environments contribute to adaptive differentiation

Historically, parental environmental effects were considered a nuisance in implementing tests of adaptation (e.g., [51]), but results from both the present and past studies [5–6, 8, 22–23, 25, 52–57] demonstrate the contribution of genetically based parental environmental effects to local adaptation. In this study, parental environmental effects contribute to a signal of adaptation in vegetative biomass where the signature of local adaptation was only apparent in saline parental environments (Fig 1A and 1B). Interestingly, the pattern of vegetative biomass when parents from both origins were grown in 0mM NaCl was similar to those observed for the parental generation [36], where all seeds used in that experiment matured under non-saline greenhouse conditions.

Given that the spatial heterogeneity in salinity is fine-scaled, that dispersal distances across these environments are small, and that dispersal across populations is high [36], theory predicts that plasticity should be favored as a mechanism for adaptation [13–14]. Moreover, because saline *M*. *truncatula* populations are a minority of populations in our study areas, we expect that plasticity could be particularly favored in saline-adapted populations. Additionally, studies on *Arabidopsis thaliana* demonstrated an increase of salinity tolerance with the number of generations of the Columbia population [23] and specific genotypes [22], which is consistent to what we observed for both reproductive traits. Thus, populations that evolved in saline environments may experience a greater tolerance to that stress as the number of generations exposed to salinity or environmental stress increases (e.g., [9]). The percent difference in reproduction between saline and non-saline populations was qualitatively larger when parental and offspring environment were matched compared to when they were mismatched (Fig 1C and 1D), consistent with the idea that population differences increase as the number of generation increases when parental and offspring environments match. Using the same genotypes of *M*. *truncatula* as in the current study, Cordeiro et al. [34] demonstrated that seed germination and timing of germination of non-saline origin populations were negatively influenced by saline environments, while saline origin populations were not affected. These early developmental differences can lead to variation in fitness and population growth rates, and increase the likelihood of parental environmental effects mediating population divergence.

The stronger effects of parental environment on traits expressed earlier in development are consistent with expectations from other studies, and are likely to contribute to population differentiation [9, 19, 55]. Using a subset of the *M*. *truncatula* genotypes used in the current study, Castro et al. [58] found that parental environmental effects altered leaf production and competitive ability. Furthermore, Vu et al. [59] found genotype-specific parental environmental effects on the expression of stored seed transcripts, which were associated with germination behavior of *M*. *truncatula* in saline conditions. Even if parental environmental effects do not persist through lifetime reproduction, they can still influence germination and survival and thus contribute to variation in fitness. Parental environmental effects on germination timing may also result in a competitive disadvantage for non-saline origin genotypes relative to saline origin genotypes, as suggested by [60]. These results suggest that multiple generations within a single environment will increase the likelihood of population differentiation between saline and non-saline environments. Overall, these patterns indicate the importance of the interplay between parental and offspring environments and on the longevity of parental effects on population adaptation.

Parental environmental effects on vegetative biomass are unlikely due to environmentally induced variation in parental provisioning, as commonly observed in other systems [19, 53, 61], because salinity treatments did not influence seed weight in either generation (S2 Table). While beyond the scope of the current study, parental modulation of offspring phenotype could be achieved by a variety of means, such as by epigenetic modification, allocation of RNA into seeds, or effects transmitted through the seed coat or other maternal tissues (e.g., as review in [8, 62]). Stored seed transcripts have been identified in this system as a potential mechanism of parental environmental effects on offspring phenotype. Recently Vu et al. [59] found support that parental exposure to saline conditions resulted in differential expression of stored seed transcripts in a small subset of *M*. *truncatula* genotypes. Furthermore, these transcripts were associated with ABA expression that influenced germination characteristics in stressful conditions, some of which are involved in DNA methylation and post-transcriptional processing of RNA [59].

### Mechanisms of salinity adaptation: avoidance and tolerance

Mechanisms that confer fitness advantages in saline environments are generally categorized as avoidance or tolerance [11] but these are not necessarily mutually exclusive [28, 32, 63]. Here we describe trait expression patterns that might entail both avoidance and tolerance mechanisms associated with salinity adaptation: phenological traits (flowering time and germination timing) associated with salinity avoidance that depended on both parental and offspring environments; and morphological and physiological traits (leaf and gas exchange) associated with salinity tolerance that depended mostly on offspring environment (Table 2). Patterns of trait expression in this study suggest that between and within generation plastic responses differentially modulate avoidance and tolerance related traits that contribute to salinity adaptation.

Studies have demonstrated that short-lived annuals exposed to stressful environments have evolved stress avoidance mechanisms that result in earlier completion of the life cycle (e.g., [23, 30, 35, 58, 60]). Tunisian *M*. *truncatula* populations demonstrate salt avoidance strategies in both early seedling and reproductive traits (i.e. germination and flowering phenology). In the parental generation of this experiment and an accompanying experiment with field collected soils, differences of two weeks in flowering time were observed between saline and non-saline genotypes [36]. Overall, between and within generation plastic responses on phenology are consistent with adaptation to environmental stress in *M*. *truncatula*.

Flowering time has been shown to be an important trait modulating local adaptation to stressful environments [25, 31–32; 64]. A leading candidate for the differences in flowering time in these populations has been identified by strong soil association and other signatures of selection: *Constans* [36], a well characterized gene involved in the control of flowering time in many angiosperms (e.g., [65]). *Constans* is a B-box regulator of the central flowering time gene *FT* in *Arabidopsis* [66]. Its regulation occurs at both mRNA and protein levels (e.g., [66–69]) and could easily regulated epigenetically in legumes, making it a candidate gene for transgenerational plasticity.

Avoidance mechanisms via earlier germination and reproduction are likely a major mechanism associated with salinity adaptation in *M*. *truncatula*, but plants must be able to grow, survive, and reproduce under salinity stress. Because of potential interactions of soil salinity with soil moisture, the ability to respond to within season plant water balance variation may be favored over adaptation to soil salinity *per se* [70]. Leaf and gas exchange traits were also consistent with expectations of greater salinity tolerance in saline origin populations and greater growth potential in non-saline origin populations. The buildup of Na+ concentration in leaves has been shown to reduce the effectiveness of carbon acquisition in plants [11, 71] and consistent with the 21.3% decline of carbon acquisition rate in 100 mM NaCl offspring environment observed in this study (Table 2).

Lower carbon acquisition rates of saline origin populations when grown in non-saline conditions suggests that salinity adaptation confers a cost in lower potential growth [69], which is also consistent with a general cost of stress tolerance [41]. However, this lower growth may be due to the tradeoff with greater water use efficiency, which is often favorable under high drought and saline conditions [40,41]. Our data suggest that the ability to maintain greater leaf water content (Fig 3D) in saline environments comes at a cost of lower photosynthetic rates (Fig 3C) as stomata remain closed to prevent water loss via evapotranspiration [11, 69], which is assumed to be beneficial under saline and drought conditions (e.g., [41]). This is similar to adaptations to a range of stressful environments (e.g., [72]) where stress-adapted populations do well in less stressful environments in the absence of competition, but lose out when they compete in these benign environments owing to costs of stress tolerance. Reduced growth in the absence of salinity stress may be a cost of salinity tolerance in *M*. *truncatula*.

The observed greater leaf water content in saline versus non-saline offspring environments (Fig 2F) may be explained by decreased stomatal conductance [37], increased concentration of osmolytes [73], or increased Na+ ions within the cytoplasm [11]. Greater leaf water content in saline environments has been proposed to allow for greater salt ion accumulation while minimizing ion toxicity [11] and to maintain carbon acquisition rates [71]. Alternatively, greater leaf water content may be a passive plastic response of increased Na+ in leaf tissue resulting in greater leaf water content. Testing this is beyond the scope of this project and would require quantifying osmolyte and Na+ ion concentrations in leaf tissue.

## Conclusion

Here we demonstrate the role of parental and offspring environments on salinity adaptation of four *M*. *truncatula* populations supporting the important contribution that past and current environmental conditions have on population differentiation. For salt adapted populations, parental growth in saline environments resulted in early completion of the life cycle (e.g., earlier germination and time to flowering), while offspring growth in saline environments resulted in salinity tolerance with the expression of traits associated with growth (leaf and gas exchange). Thus, directly manipulating past and current environmental conditions may highlight the importance of within and between generation environmental responses to environmental stress in other systems. Results by Suter and Widmer [23] and those presented here suggests that tolerance to soil salinity may increase as the number of generations experiencing salinity increases, consistent with an environment by environment interaction that occurs between generations. In this system, salinity adaptation is associated with a cost of lower growth potential when salinity stress is removed, thus preventing genotypes from saline adapted populations from successfully dispersing into non-saline environments. The complexity of salinity adaptation may also result from differences in plant densities, as shown by Castro et al., [58], which demonstrated that non-tolerant genotypes of *M*. *truncatula* may benefit in mixed stands with tolerant genotypes in saline environments due to competitive effects. Overall, offspring’s responses to stress may be modulated by a combination of transgenerational and within generation environmental effects; such transgenerational environmental effects can contribute to adaptive evolution rather than be a source of statistical noise.

## Supporting Information

S1 Fig Population least square means and one standard error for days to first flowering. Means for non-saline origin populations are shown with open symbols and means for saline origins are shown with closed symbols.
(TIF)

S1 File Complete dataset used for the offspring generation. Sheet one of the spreadsheet contains complete explanations for the column headings used for each column of data in sheet 2. The parental generation data is analyzed and described in [36].
(XLSX)

S1 Table List of *Medicago truncatula* (Fabaceae) accessions from the two saline-origin populations (TN1 and TN8) and two non-saline origin populations (TN7 and TN9) used in the experiment to quantify parental and offspring salinity effects on offspring phenotype and performance. Asterisks indicate the subset of genotypes where carbon acquisition rates were measured.
(DOCX)

S2 Table Analysis of seed weight during the parental and offspring generation for *Medicago truncatula*. During the first generation, for each genotype seeds were collected from pods that were produced at peak maturation time. During the second generation, for each genotype seeds were collected from pods collected and analyzed in the experiment. Viable seeds were counted and weighed to the nearest 0.01mg. Mixed-model ANOVA was performed on average seed weight including origin, population, salinity treatment (Generation 1 included only offspring environment; Generation 2 included parental and offspring environment) with genotype included as a random effect. Origin of population, population, parental environment and offspring environment were treated as fixed effects and F-values are reported. Genotype was treated as a random effect and χ^2^ values are reported.
(DOCX)
