# Supplementary material for: Salinity Adaptation and the Contribution of Parental Environmental Effects in Medicago truncatula
Source: PLoS One. 2016 Mar 4;11(3):e0150350. doi: 10.1371/journal.pone.0150350 (PMC4778912; doi:10.1371/journal.pone.0150350)
Supplement: S1 Table — Asterisks indicate the subset of genotypes where carbon acquisition rates were measured. (DOCX) [file pone.0150350.s003.docx]

Supplemental Table and Figure legends.

S1. Supplemental Table S1. List of *Medicago truncatula* (Fabaceae) accessions from the two saline-origin populations (TN1 and TN8) and two non-saline origin populations (TN7 and TN9) used in the experiment to quantify parental and offspring salinity effects on offspring phenotype and performance. Asterisks indicate the subset of genotypes where carbon acquisition rates were measured.

| Enfidha (TN1)  Saline Soils | Soliman (TN8)  Saline Soils | El Kef (TN7)  Non-saline Soils | Bulla Regia (TN9)  Non-saline Soils |
| --- | --- | --- | --- |
| 1.1  1.3  1.5  1.11*  1.13  1.15  1.16*  1.17*  1.18*  1.21 | 8.3  8.4*  8.5  8.15*  8.21  8.22  8.23*  8.24*  8.25  8.28 | 7.2  7.4  7.11  7.13*  7.17  7.19  7.20  7.22*  7.23* | 9.3  9.4  9.5  9.12*  9.15  9.17  9.20  9.21*  9.22  9.24* |
