# Supplementary material for: Salinity Adaptation and the Contribution of Parental Environmental Effects in Medicago truncatula
Source: PLoS One. 2016 Mar 4;11(3):e0150350. doi: 10.1371/journal.pone.0150350 (PMC4778912; doi:10.1371/journal.pone.0150350)
Supplement: S2 Table — During the first generation, for each genotype seeds were collected from pods that were produced at peak maturation time. During the second generation, for each genotype seeds were collected from pods collected and analyzed in the experiment. Viable seeds were counted and weighed to the nearest 0.01mg. Mixed-model ANOVA was performed on average seed weight including origin, population, salinity treatment (Generation 1 included only offspring environment; Generation 2 included parental and offspring environment) with genotype included as a random effect. Origin of population, population, parental environment and offspring environment were treated as fixed effects and F-values are reported. Genotype was treated as a random effect and χ2 values are reported. (DOCX) [file pone.0150350.s004.docx]

S2. Table. Analysis of seed weight during the parental and offspring generation for *Medicago truncatula*. During the first generation, for each genotype seeds were collected from pods that were produced at peak maturation time. During the second generation, for each genotype seeds were collected from pods collected and analyzed in the experiment. Viable seeds were counted and weighed to the nearest 0.01mg. Mixed-model ANOVA was performed on average seed weight including origin, population, salinity treatment (Generation 1 included only offspring environment; Generation 2 included parental and offspring environment) with genotype included as a random effect. Origin of population, population, parental environment and offspring environment were treated as fixed effects and F-values are reported. Genotype was treated as a random effect and χ^2^ values are reported.

| Source | Num. df | Den. df | F-value or χ^2^ value | P-value |
| --- | --- | --- | --- | --- |
| Generation 1  Origin  Population (Origin)  Salinity Treatment  Origin X Salinity  Pop (Origin) X Salinity  Genotype (Origin Pop) | 1  2  1  1  2  1 | 36  36  33  33  33 | 0.86  0.75  2.29  0.60  2.18  3.6 | 0.3597  0.4797  0.1396  0.4451  0.1292  0.0577 |
| Generation 2  Origin  Population(Origin)  Parental Environment  Offspring Environment  Origin X Parental Environment  Origin X Offspring Environment  Pop (Origin) X Parental Environment  Pop(Origin) X Offspring Environment  Parental Environment X Offspring Environment  Origin X Parental Env. X Offspring Env.  Pop(Origin) X Parental Env. X Offspring Env.  Genotype (Origin Pop) | 1  2  1  1  1  1  2  2  1  1  2  1 | 36  36  108  108  108  108  108  108  108  108  108 | 13.60  4.46  0.55  0.92  0.08  0.26  0.61  0.26  0.00  0.07  0.40  75.4 | 0.0007  0.0185  0.4616  0.3400  0.7825  0.6111  0.5434  0.7712  0.9547  0.7919  0.6729  <0.0001 |
